# DNA Methylation Analysis to Unravel Altered Genetic Pathways Underlying Early Onset and Late Onset Neonatal Sepsis. A Pilot Study

**DOI:** 10.3389/fimmu.2021.622599

**Published:** 2021-02-15

**Authors:** Sheila Lorente-Pozo, Paula Navarrete, María José Garzón, Inmaculada Lara-Cantón, Jesús Beltrán-García, Rebeca Osca-Verdegal, Salvador Mena-Mollá, Eva García-López, Máximo Vento, Federico V. Pallardó, José Luis García-Giménez

**Affiliations:** ^1^Neonatal Research Group, Health Research Institute La Fe, Valencia, Spain; ^2^Division of Neonatology, University and Polytechnic Hospital La Fe, Valencia, Spain; ^3^EpiDisease S.L. (Spin-off From the CIBER-ISCIII), Parc Científic de la Universitat de València, Paterna, Spain; ^4^Centro de Investigación Biomédica en Red de Enfermedades Raras (CIBERER), Valencia, Spain; ^5^Department Fisiología, Facultad de Medicina y Odontología, Universidad de Valencia-INCLIVA, Valencia, Spain

**Keywords:** neonatology and pediatric intensive care, DNA methylation, sepsis, inflammation, immunosuppression

## Abstract

**Background:** Neonatal sepsis is a systemic condition widely affecting preterm infants and characterized by pro-inflammatory and anti-inflammatory responses. However, its pathophysiology is not yet fully understood. Epigenetics regulates the immune system, and its alteration leads to the impaired immune response underlying sepsis. DNA methylation may contribute to sepsis-induced immunosuppression which, if persistent, will cause long-term adverse effects in neonates.

**Objective:** To analyze the methylome of preterm infants in order to determine whether there are DNA methylation marks that may shed light on the pathophysiology of neonatal sepsis.

**Design:** Prospective observational cohort study performed in the neonatal intensive care unit (NICU) of a tertiary care center.

**Patients:** Eligible infants were premature ≤32 weeks admitted to the NICU with clinical suspicion of sepsis. The methylome analysis was performed in DNA from blood using Infinium Human Methylation EPIC microarrays to uncover methylation marks.

**Results:** Methylation differential analysis revealed an alteration of methylation levels in genomic regions involved in inflammatory pathways which participate in both the innate and the adaptive immune response. Moreover, differences between early and late onset sepsis as compared to normal controls were assessed.

**Conclusions:** DNA methylation marks can serve as a biomarker for neonatal sepsis and even contribute to differentiating between early and late onset sepsis.

## Introduction

Neonatal sepsis is a leading cause of mortality and morbidity in the neonatal period, especially for preterm infants (1). Sepsis is defined as a dysregulated host response caused by infection which can trigger a life-threatening organ failure (2). However, neonatal sepsis lacks a consensus definition (3) and therefore a full understanding of the underlying mechanisms which are functionally distinct in children and adults is needed (3). The neonatal immune response depends mainly on the innate immune system, which is underdeveloped (4, 5); along with a predominant hypo-inflammatory response, this can lead to immunosuppression (6).

Diagnosis and prognosis of neonatal sepsis is a challenge because clinical signs and symptoms are often non-specific. In addition, the result of the blood culture for confirmation of the diagnosis is frequently delayed or provides no results (7). Moreover, given the effects of sepsis in preterm infants on long-term growth and development, efforts to reduce the rates of infection in the neonatal intensive care unit (NICU) are among the most important interventions in neonatal care (8). According to the onset of age, neonatal sepsis is divided into early-onset sepsis (EOS) and late-onset sepsis (LOS). EOS is defined as the onset of features of sepsis that appear within 72 h of life caused by microorganisms acquired from the mother's genital tract during delivery (9–11). In contrast, LOS is defined as onset of features of sepsis appear after 72 h of life due to nosocomial infections acquired during hospitalization (11, 12).

In this context, there is a growing interest in understanding the role of epigenetics in the maturation of the immune system during early life (13) and particularly in sepsis (14, 15). Epigenetics encompasses all the mechanisms that control the gene expression pattern without altering the DNA sequence itself (16–18). Changes in the epigenome can impair immune response to infections (14, 15), suggesting that the study of epigenetic traits through epigenome-wide association studies (EWAS) can improve our understanding of sepsis onset and progression, and even short- and long-term consequences of the disease (19).

In particular, DNA methylation (DNAm) is an important regulator of the immune system (20), maintaining fine-tuned immunological mechanisms needed for an adequate host defense. One of the major consequences of neonatal sepsis is long-term persistence of immunosuppression related to epigenetic dysregulation (6), leading to subsequent infections and increased risk of mortality (15, 21). Importantly, DNAm may drive sepsis-induced immunosuppression through the epigenetic reprogramming of hematopoietic progenitor cells (19). In line with this, although DNAm changes in adult sepsis have been described in some studies (22, 23), information on neonatal sepsis is lacking (24).

We aimed to detect changes in DNAm signatures in preterm neonates that might provide an insight into molecular events driving neonatal sepsis. Consequently, this could contribute to the identification of new biomarkers for the diagnosis and prognosis of neonatal sepsis.

## Materials and Methods

### Experimental Design

The study was performed in the NICU of the Hospital Universitario y Politécnico La Fe (Valencia, Spain). Samples from neonates were obtained from the study approved by the Ethics Committee with registration number 2017/0470. Parents of all the patients signed the informed consent.

This is a prospective cohort study that included preterm neonates born at ≤32 weeks of gestation age. Inclusion and exclusion criteria for the study are shown in Supplementary Table 1.

Samples from 23 preterm infants were collected coincident with clinical suspicion of EOS or LOS. Preterm neonates with similar gestational and postnatal ages and perinatal characteristics but free of infection and without suspicion of sepsis acted as controls. EOS and LOS were diagnosed by attending staff neonatologists in the NICU. Patients who met EOS and LOS criteria were included in the study (21, 22). Six patients had confirmed EOS, nine patients confirmed LOS, and two patients suffered both types. Six non-septic preterm neonates were used as controls (Table 1). Although a positive blood culture is the golden standard for the diagnosis of sepsis including EOS and LOS, often blood culture renders negative due to the low blood volume available and contamination by peripheral colonizing bacteria. Therefore, in clinical neonatology there is a distinction between “culture positive sepsis” and “clinical sepsis” (Table 2). Culture positive sepsis is characterized by a positive bacterial growth in two blood samples taken under stringent aseptic conditions and is treated with specific antibiotics provided by the antibiogram. Clinical sepsis is characterized by the presence of evident of clinical signs that can be or not accompanied by changes in acute phase reactant biomarkers such as CRP, IL-6, or PCT, and or positive peripheral smears and is treated with an empirical combination of antibiotics according to the bacterial eco-system of the NICU. These criteria were not mutually exclusive, and some patients were identified as suffering from both EOS and LOS, being the blood sample taken for analysis after the second sepsis episode.

**Table 1:** Perinatal characteristics of preterm infants ≤32 weeks of gestation with suspicion or confirmed sepsis (EOS, LOS, and EOS+LOS) and neonates without sepsis as controls.

**Variables**	**LOS** **(*N* = 9)**	**EOS** **(*N* = 6)**	**LOS and EOS** **(*N* = 2)**	**CRL** **(*N* = 6)**	***p*-value** ** CRL vs. LOS**	***p*-value** ** CRL vs. EOS**	***p*-value** ** CRL vs. LOS+EOS**	***p*-value** ** EOS vs. LOS**
Gestational age (weeks) (median; 5–95% CI)	26 (25–29)	27 (25–30)	25 (25)	29.5 (28–30)	^*^0.02^a^	0.45^a^	^*^0.04^a^	0.40^a^
Birth weight (g) (mean ± SD)	797.8 ± 228.7	1082.5 ± 338.2	845 ± 49.5	1253 ± 261	^*^0.02^a^	0.68^a^	0.26^a^	0.21^a^
Apgar score 1 min (median; 5–95% CI)	7 (4–8)	7 (4–9)	5 (4–6)	8.5 (6–10)	0.27^a^	0.59^a^	0.22^a^	0.97^a^
Apgar score 5 min (median; 5–95% CI)	9 (8, 9)	8.5 (6–10)	7.5 (7, 8)	9 (8–10)	0.69^a^	0.70^a^	0.25^a^	>0.99^a^
Male sex (*n*, %)	7 (77.8)	4 (66.7)	2 (100)	3 (31)	0.33^b^	>0.99^b^	0.46^b^	>0.99^b^
Type of delivery: vaginal/cesarean (*n*, %)	2/7 (22/78)	0/6 (0/100)	2/0 (100/0)	1/5 (17/83)	>0.99 ^b^	>0.99^b^	0.11^b^	0.49 ^b^
Twin pregnancy (*n*, %)	4 (44.44)	3 (31)	2 (100)	3 (31)	>0.99 ^b^	>0.99^b^	0.46 ^b^	>0.99 ^b^
Antenatal steroids full course^α^ (*n*, %)	9 (100)	6 (100)	2 (100)	6 (100)	>0.99 ^b^	>0.99^b^	>0.99 ^b^	>0.99 ^b^
Chorioamnionitis^β^ (*n*, %)	1 (11.11)	1 (16.67)	1 (31)	0 (0)	>0.99^b^	>0.99^b^	0.25^b^	>0.99^b^
Antibiotic therapy to mother (*n*, %)	3 (33.33)	6 (100)	6 (100)	0 (0)	0.23^b^	^*^ <0.01^b^	^*^ <0.01^b^	^*^0.03^b^
Weight at sample collection (g) (mean ± SD)	1087 ± 270.2	1007 ± 266.4	1223 ± 661.1	1205 ± 243.4	0.87^a^	0.66^a^	>0.99^a^	0.95^a^

α *Full course of antenatal steroids: 12-mg doses of betamethasone given intramuscularly 24 h apart or four 6-mg doses of dexamethasone administered intramuscularly every 12 h*.

β *Chorioamnionitis or intra-amniotic infection was defined as an acute inflammation of the membranes and chorion of the placenta with clinical, histologic, or microbiological findings*.

a *Tukey's multiple comparisons test*.

b *Fisher's exact test*.

* *p-value < 0.05 was considered statistically significant*.

**Table 2:** Summary of microbiological, clinical, and analytical results in patients with early and late onset sepsis.

**Patient identification**	**Blood culture results**	**Clinical signs**	**Dab suspected sepsis**	**Lactic acid**	**CRP**	**IL6**	**WBC count (per mm^**3**^)**	**Neutrophils (per mm^**3**^)**
**Late onset sepsis**								
Patient 1 (5)	*Staphylococcus epidermidis*	Present	7	2.4	75.9	–	41,250	38,130
Patient 2 (7)	*S. epidermidis*	Present	19	1.5	31.9	–	16,840	8,450
Patient 3 (19)	*S. epidermidis*	Present	25	3.6	64.6	–	35,510	19,630
Patient 4 (20)	*S. epidermidis*	Present	26	1.1	13.7	–	16,220	3640
Patient 5 (23)	*S. epidermidis*	Present	11	1.3	8.3	–	28,750	18,470
Patient 6 (14)	*Staphylococcus aureus*	Present	6	2.5	58.8	–	8,440	6,650
Patient 7 (25)	*S. aureus*	Present	>3	1.9	1.3	–	2,630	820
Patient 8 (32)	*S. aureus*	Present	>3	3.7	14.9	–	5,080	2,270
Patient 9 (33)	Negative	Present	11	2.0	36	–	23,590	19,120
**Early onset sepsis**								
Patient 10 (8)	Negative	Present	<1	1.2	32.2	–	27,170	21,150
Patient 11 (30)	Negative	Present	<1	1.6	2.1	1,180	6,580	1,280
Patient 12 (34)	Negative	Present	<1	2.3	11.7	–	7,950	4,650
Patient 13 (17)	Negative	Present	<1	1.6	15.2	306	14,300	6,120
Patient 14 (35)	Negative	Present	2	1.3	4.2	–	9,580	5,440
Patient 15 (36)	*Staphylococcus haemolyticus*	Present	<1	1.6	10.8	–	9,150	7,400
**Early and Late onset Sepsis**								
Patient 16 (4)	*Escherichia coli* *S. epidermidis*	Present Present	<1 5	2.7	37.3	–	35,560	25,420
Patient 17 (37)	*S. epidermidis* *S. epidermidis*	Present Present	<1 17	1.3	111	–	10,500	7,750

**Figure 1:**
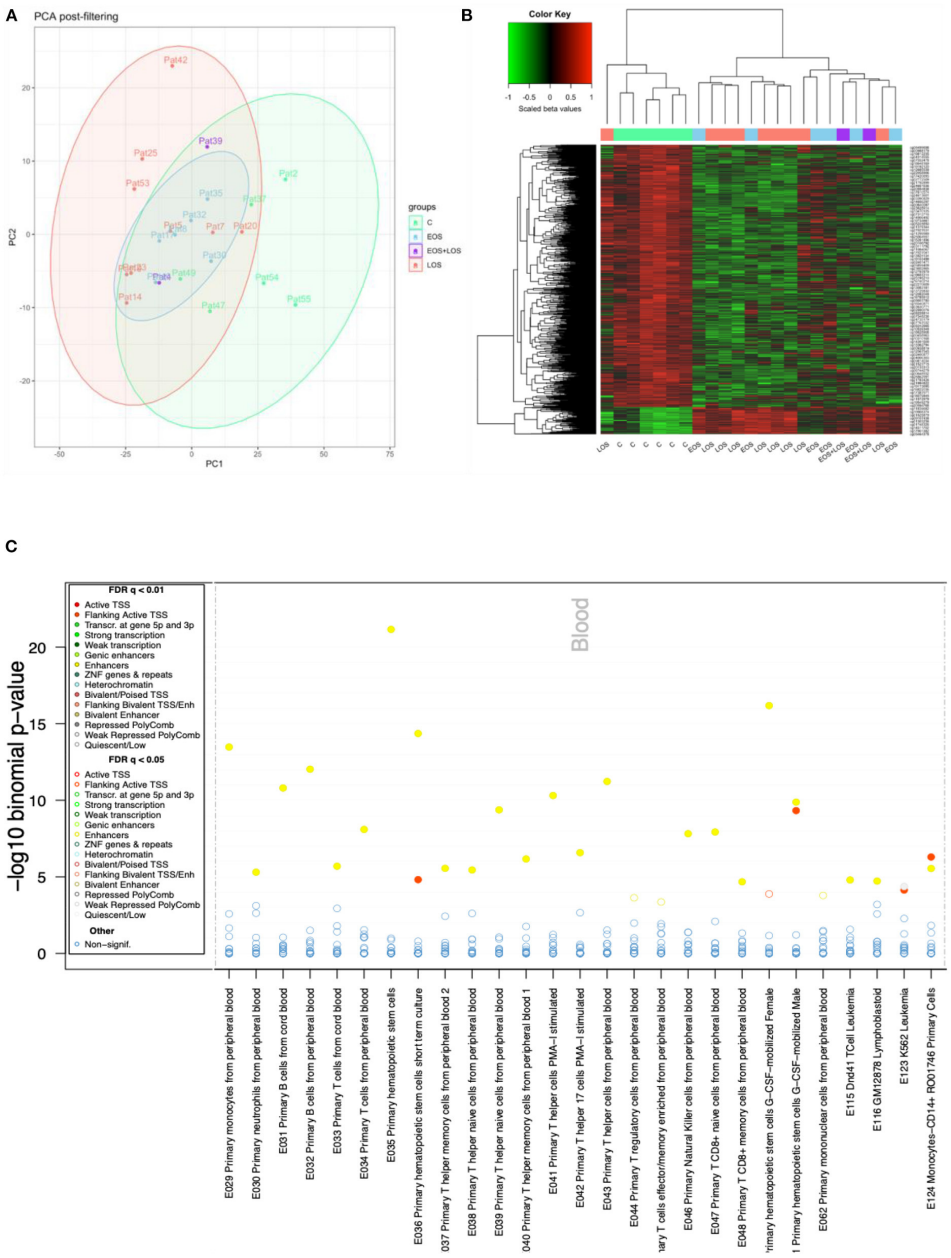
DNAm analysis. **(A)** Principal component analysis (PCA) of methylation data of control (light green), LOS (pink), EOS (light blue), and EOS plus LOS (purple) samples. **(B)** DNAm heatmap showing the significant top 1,000 differentially methylated CpGs between controls and neonates with sepsis, obtained with limma. The methylation metric used to obtain the heatmap is the beta values scaled by the median, shown at the top left color scale (where red represents higher methylation levels, and green lower methylation levels). This displays the clustering of sepsis samples separated from control samples with the exception of one LOS sample. **(C)** eFORGE analysis. Enrichment analysis of regulatory element classes in different blood cell types for the top 1,000 CpG sites between septic neonates and control neonates. Cell types are shown on the horizontal axis and the significance (–log_10_ binomial *p*-values) is shown on the vertical axis. Each dot represents an enrichment q-value for a given cell type, colored according to the enriched regulatory element class, as shown in the legend (where yellow points correspond to enhancers and red points correspond to transcription start sites).

*Dab: days after birth; CRP, C-reactive protein; WBC, white blood cell count; IL-6, interleukin 6. When clinical signs of sepsis were present, the diagnosis of sepsis was confirmed. Sign (–) indicates no measured parameter. Patient number noted using parentheses corresponds to the patients showed in PCA graph in Figure 1*.

### Blood Sampling and DNA Extraction

Blood (0.5 mL) was sampled from a peripheral vein or central catheter using a heparinized syringe. Blood was centrifuged (1,500 × g for 10 min) at 4°C to separate plasma from the cell pellet. Cell fractions were stored at −80°C until processing. Total DNA was isolated from the cell pellet with All-In-One DNA/RNA Miniprep Kit (BS88203, Bio Basic Canada Inc., Canada) following the manufacturer's instructions. Concentration and purity of DNA was determined with the fluorometric method (Quant-iT PicoGreen dsDNA Assay, Life Technologies, Carlsbad, CA, USA).

### DNA Methylation Profiling Using Illumina EPIC 850K Array

The measurement of genome-wide methylation on the 23 samples was performed by means of the Infinium Human DNA Methylation EPIC 850K arrays (Illumina Inc, San Diego, CAL, USA) following the manufacturer's instructions.

### Bioinformatic Analysis

The minfi R-package (27) was used to process and normalize the arrays (28, 29). The identification of differentially methylated CpGs (DMCs) was performed using limma (30) using an FDR cutoff of 0.05, which is the expected proportion of errors and controls for a low proportion of false positives (38). With the aim of discovering differentially methylated regions (DMRs), we employed two complementary bioinformatic tools: DMRcate (34) and mCSEA (39) R-packages. We performed the DNAm differential analysis between the groups of the study. Finally, we performed the overlap of both DMR sets.

DMRs were functionally enriched in both gene ontology (GO) terms and KEGG pathways by means of an over-representation analysis (ORA) using the clusterProfiler R-package (40). The selected FDR threshold was 0.05. Furthermore, the top 1,000 DMCs were analyzed for cell-type enrichment using eFORGE (35), which determines the cell type-specific regulatory component of a set of EWAS-identified differentially methylated positions to identify disease-relevant cell types in a specific disease.

### Statistical Analysis

Quantitative variables were analyzed for normality using the Kolmogorov-Smirnov test, and an ordinary one-way ANOVA was performed for multiple comparisons using Tukey's test. Furthermore, qualitative variables were analyzed using Fisher's exact test.

## Results

### Neonatal Characteristics and Microbiological Data

Twenty-three preterm infants (≤32 weeks of gestation age) were included. Out of these, 17 were diagnosed of neonatal sepsis cases and 6 pre-term neonates were selected as non-septic controls. Subjects' characteristics are shown in Table 1 and the criteria for “culture positive sepsis” and “clinical sepsis,” as well as inflammatory biomarkers, are shown in Table 2. Hence, 9 subjects developed LOS, 6 subjects developed EOS, 2 subjects developed EOS+LOS, and 6 subjects were considered healthy controls. In Table 1, we found statistical differences in the gestational age when comparing LOS [26 (25–29) weeks] and EOS+LOS [25 (25) weeks] to non-septic pre-term neonates [29.5 (28–30) weeks]. Moreover, we also found statistical differences in birth weight between neonates who developed LOS (797.8 ± 228.7 g) and pre-term neonates (1,253 ± 261 g), and in the antibiotic therapy to mother when comparing EOS to the other clinical groups (See Table 2).

The blood culture results are presented in Table 2 and Supplementary Figure 1. In one case of LOS and five cases of EOS, it was not possible to identify the microorganism.

### Differential Methylation Profiles Between Neonatal Sepsis Subtypes and Control Subjects

Principal component analysis (PCA) of the processed data showed a slight separation between septic and control samples, with some overlap. Regarding subtypes, LOS samples showed greater separation from controls than did EOS (Figure 1A).

The differential methylation analysis between septic and control neonates showed 77,380 differentially methylated CpGs (DMCs). The top 1,000 DMCs were plotted in a heatmap showing a similar methylation pattern in both clinical groups, EOS and LOS (Figure 1B). The eFORGE analysis of the top 1,000 DMCs revealed enrichment in blood enhancers, particularly primary hematopoietic stem cells (Figure 1C).

**Figure 2:**
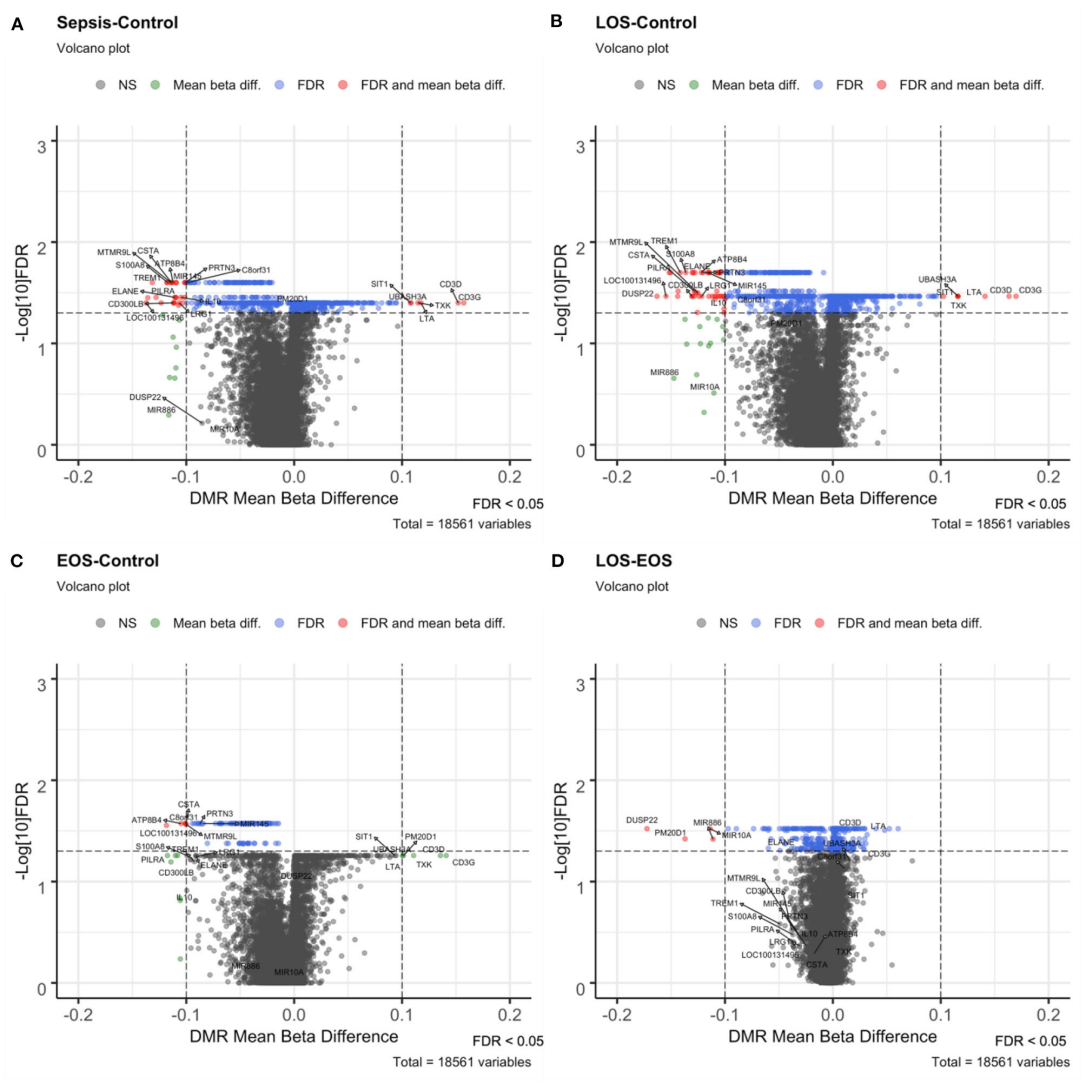
Volcano plots of the different contrasts performed with mCSEA. Volcano plots show the beta-value difference in the x-axis plotted vs. –log_10_ FDR in the y-axis. Black (not significant) and green (Mean beta diff.) dots were non-significant promoters, while blue (FDR) and red (FDR and mean beta diff.) dots correspond to the significant DMRs, below the selected FDR threshold of significance set at 0.05. Those with a mean beta difference >10% are represented as red and green dots**. (A)** Volcano plot of the sepsis vs. control contrast, **(B)** LOS vs. control contrast, **(C)** EOS vs. control comparison, and **(D)** LOS vs. EOS.

To identify DMRs, we used the mCSEA approach, testing 1,051 promoters and 119 genes with differential methylation levels in the neonatal sepsis condition vs. controls. In particular, we found 437 hypomethylated promoters, 26 of which exhibited a beta-value difference >10% (including the promoters for *EGOT, IL10, CPT1B, PILRA, ELANE, TREM1, PRTN3, MIR145, S100A8, CSTA, MS4A3, ATP8B4, CPT1B, CMYA5, LRG1*, and *CD300LB)* and 614 hypermethylated promoters, 6 of which exhibited a beta-value difference >10% for *CD3G, CD3D, LTA, TXK, UBASH3A*, and *SIT1* genes (Figure 2A). Conversely, in contrast with the high number of promoters with differential hypomethylation, only 4 genes (*MPO, LOC100131496, SPI1*, and *PLEK*) showed a beta-value difference above 10%.

In addition, we used the DMRcate method and identified 14,846 genomic regions by fitting DMCs spatially. The vast majority of DMRs were hypomethylated regions, and only 1,577 were hypermethylated. Furthermore, those with a mean beta difference above 10% provided 1,474 DMRs with hypomethylation and 386 DMRs with hypermethylation.

After overlapping the DMR sets obtained with both methods (mCSEA and DMRcate), 302 promoters and 108 genes were found to be hypomethylated and 143 promoters hypermethylated. Table 3 shows the promoters with the greatest beta differences (>10%) obtained with the two methods for the comparisons between neonatal sepsis and control neonates.

**Table 3:** General overview of differentially methylated (hypomethylated and hypermethylated) promoters detected by both DMR detection methods (mCSEA and DMRcate) for the comparison between neonatal sepsis and control neonates, including mean beta-value difference, chromosome location, and a brief description.

**Methylation status**	**Gene**	**Localization**	**Mean beta difference**	**Description**
Hypo-	S100A8	chr1	−0.11720	Inflammatory mediator participating in the amplification of the immune response. It has also been proposed as a mediator of immunosuppressive states by regulating myeloid-derived suppressor cells (MDSCs)
	CSTA	chr3	−0.11370	Cysteine protease inhibitor (cystatin superfamily) which plays a role in epidermal development and maintenance. It is expressed in tissues participating in the first-line defense against pathogens
	ATP8B4	chr15	−0.11232	Member of type IV P-type ATPases which catalyze the translocation of phospholipids in the cell membrane
	TREM1	chr6	−0.11815	Cell surface receptor that stimulates the release of pro-inflammatory cytokines and chemokines, and cell surface activation molecules in neutrophils and monocytes. TREM1 amplifies inflammation and is a crucial mediator of septic shock
	CD300LB	chr17	−0.11186	Activating receptor of the immunoglobulin superfamily expressed on myeloid cells
	ELANE	chr19	−0.11067	Serine protease involved in the extracellular matrix degradation of microorganisms through the proteolysis of elastin and other components
	CMYA5	chr5	−0.10973	Cardiomyopathy-associated protein 5
	LRG1	chr19	−0.10656	Expressed during neutrophilic granulocyte differentiation, involved in neutrophil degranulation
	IL10	chr1	−0.10504	Anti-inflammatory cytokine involved in immunoregulation and inflammation
	MIR145	chr5	−0.10161	miRNA which plays a role in the growth and division of megakaryocytes
	PRTN3	chr19	−0.10104	Serine protease, paralog of neutrophil elastase (ELANE)
Hyper-	CD3G	chr11	0.15725	Subunit of the TCR-CD3 complex, on the surface of T-cells
	CD3D	chr11	0.15212	Subunit of the TCR-CD3 complex, on the surface of T-cells
	LTA	chr6	0.11763	Cytokine that mediates in inflammatory, immunostimulatory, and antiviral responses
	TXK	chr4	0.11473	Tec family protein tyrosine kinase that regulates T-cell function and differentiation, and participates in Th1 cytokine production
	UBASH3A	chr21	0.10833	Encodes a protein that belongs to the T-cell ubiquitin ligand (TULA) that negatively regulates T-cell signaling
	SIT1	chr9	0.10786	Negatively regulates TCR-mediated signaling in T-cells, and is involved in positive selection of T-cells

### Differential Methylation in EOS and LOS to Identify Distinct Molecular Responses in Both Subtypes of Neonatal Sepsis

As shown in the results above, comparisons between EOS and controls and between LOS and EOS exhibited smaller differences in DNA methylation between conditions. Therefore, we used the mCSEA method which allows discovery of regions with high sensitivity and subtle differences in DNAm but with consistency across a genomic region.

Among the 1,433 promoters obtained with mCSEA, 50 had a difference >10%, and some of them were associated with relevant genes involved in immunological functions (Figure 2B). Nevertheless, the comparison between EOS and controls showed differential methylation in only 44 genes and 108 promoters, in which relevant genomic regions that showed significant changes in LOS—such as *S100A8, TREM1, ELANE, CD3D*, and *CD3G*—were not present. Importantly, promoters of *CSTA, MIR145, PRTN3*, and *ATP8B4* genes were differentially methylated in EOS as well as in LOS when compared to controls (Figure 2C).

When we compared LOS with EOS, 803 differentially methylated promoters were obtained, most of them with small differences except for 4 hypomethylated promoters, which displayed large beta differences for gene promoters of *DUSP22, PM20D1, MIR10A*, and *MIR886* (Figure 2D).

### Pathways Related With Immune Response Are Altered by DNA Methylation in Neonatal Sepsis

**Figure 3:**
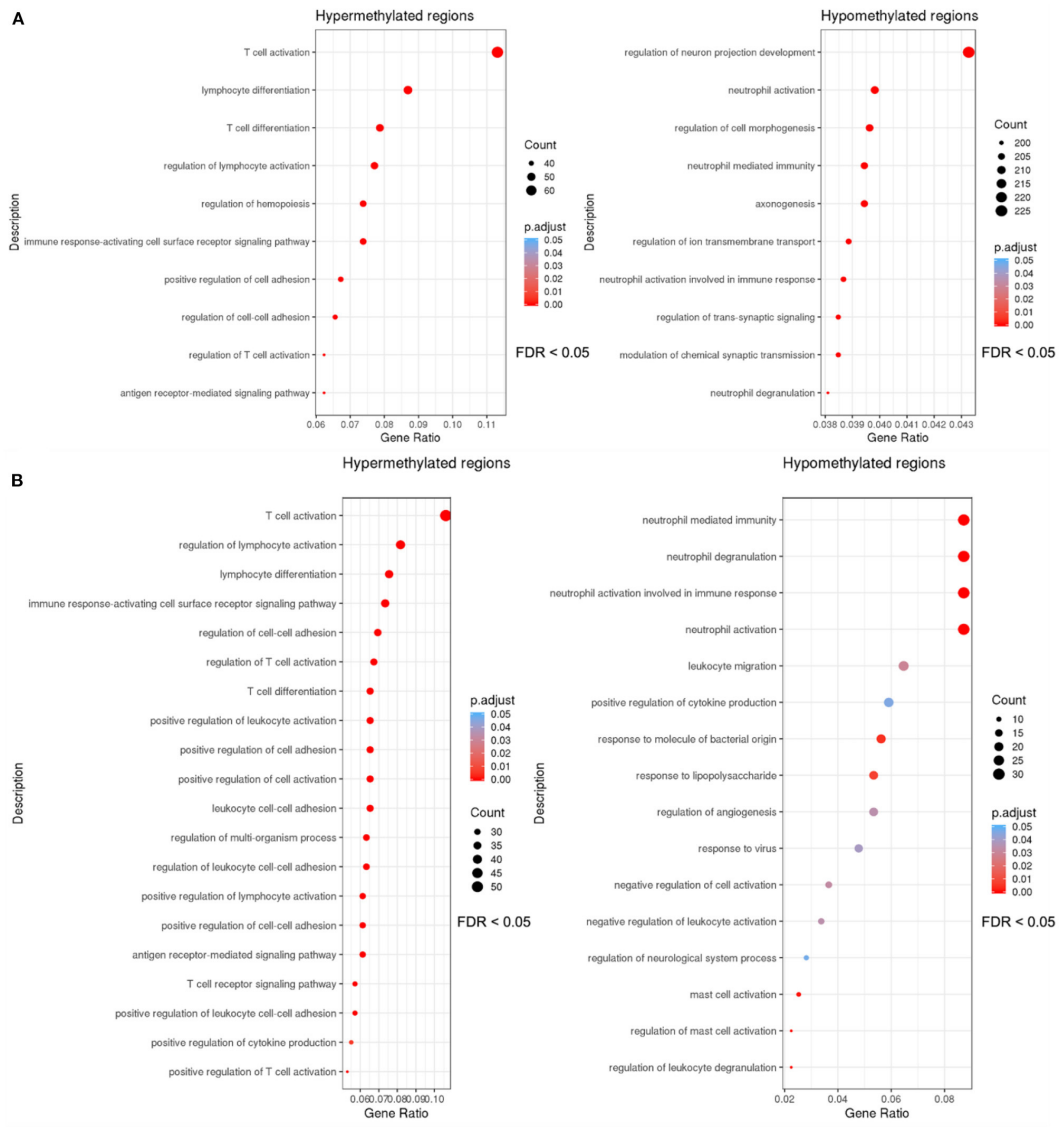
Dotplots of the gene ontology-biological process terms (GO-BP) overrepresented with an FDR threshold of <0.05 in the sets of DMRs. The y axis represents the significant GO terms with the higher proportion of DMRs of the set involved, and the gene ratio in the x-axis represents these proportions (number of DMRs involved in each GO term over the total number of DMRs in the set). The size of each dot or gene cluster depends on the gene count that contributes to the enrichment of that pathway, and the adjusted *p*-value obtained for the terms with the Fisher's exact test in ORA determines the color of the dots. **(A)** Dotplot of the biological processes in which the DMRs (promoters) obtained with mCSEA are enriched. **(B)** Dotplot of the enrichment results of the DMR set (genes and promoters) obtained with DMRcate.

DMR sets obtained using mCSEA and DMRcate methods for sepsis vs. control comparison were enriched to detect important functions, and as a result similar GO biological processes related with immune response were obtained using both methods (Figure 3).

**Figure 4:**
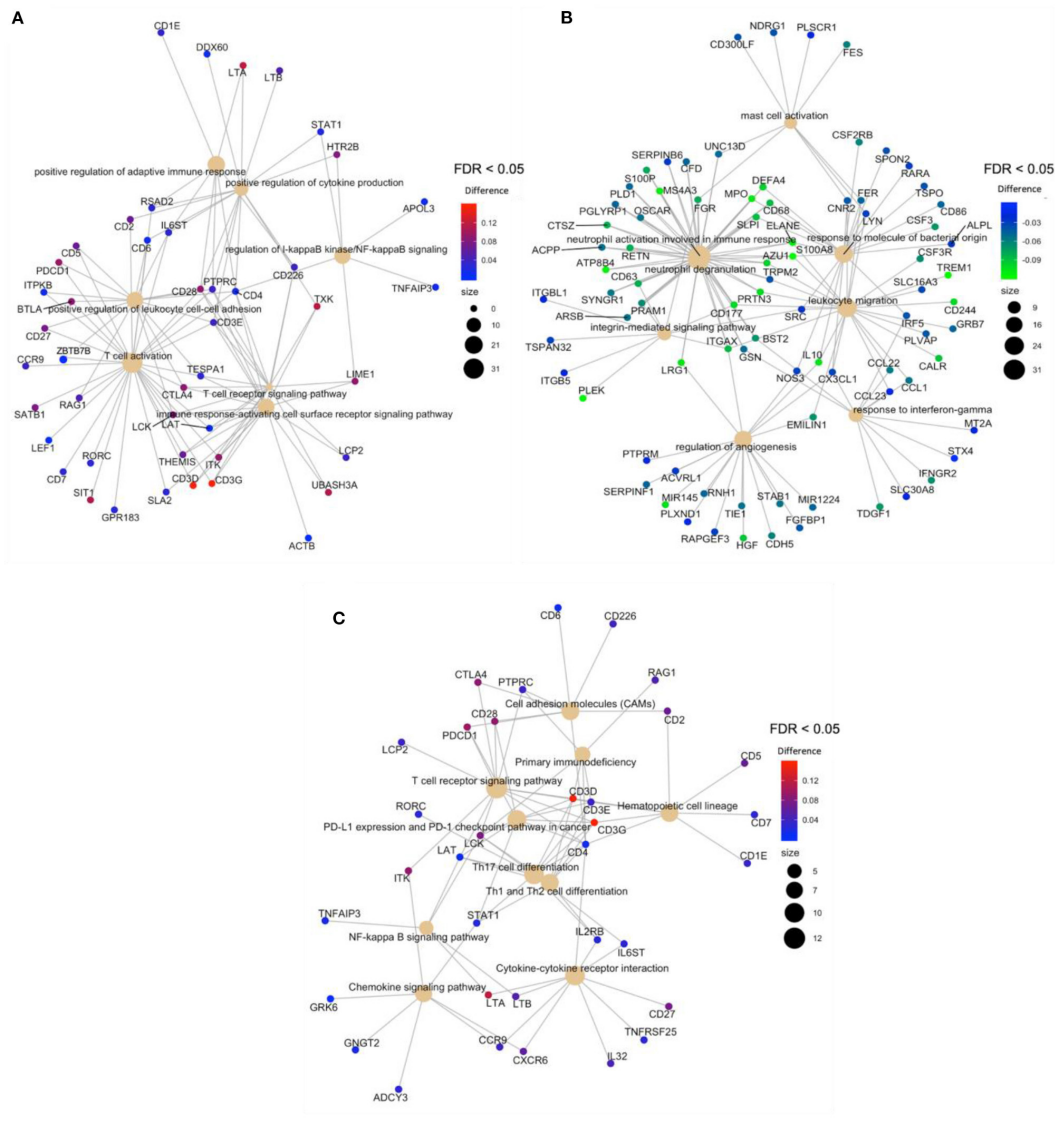
Gene-concept network (cnetplot) of overlapping DMRs. It shows the enriched concepts, obtained with an FDR cut-off of 0.05 (GO biological processes or KEGG pathways) linked to the DMRs involved in each concept, and the relation between them when genes are involved in more than one process. The size of the concept nodes depends on the gene count involved in that pathway or process, while the color of the gene nodes depends on their methylation level according to the displayed color gradient. **(A)** Cnetplots of GO terms of hypermethylated regions in neonatal sepsis, **(B)** GO terms of hypomethylated regions in neonatal sepsis, and **(C)**. KEGG pathways of hypermethylated regions.

After the overlapping of the DMRs for genes and promoters, obtained with mCSEA and DMRcate approaches, an overrepresentation analysis (ORA) revealed the enrichment of hypermethylated regions in T-cell activation and T-cell differentiation to be the processes with the highest significance, among other relevant GO terms (Figure 4A), which demonstrates the scarce immunoreactivity of the cells in septic neonates. Moreover, a high proportion of hypomethylated regions were enriched in neutrophil degranulation and activation involved in immune response processes, as well as in mast cell activation and response to molecules of bacterial origin (Figure 4B). Regarding KEGG pathways, the most relevant pathways enriched in the set of hypermethylated DMRs were T-cell receptor signaling pathway, Th17 cell differentiation, and PD-L1 expression and PD-1 checkpoint pathway, which are related with immunosuppression processes (Figure 4C).

## Discussion

Sepsis produces high mortality and morbidity and adverse long-term effects in neonates. The pathophysiology of neonatal sepsis is not yet fully understood (3). We propose that epigenetics is involved in the immunopathology of sepsis and can provide biomarkers for clinical management of early onset and late onset sepsis.

Neonatal immune development is complex and incompletely understood. Moreover, neonatal immune development is orchestrated by many factors including intra- and extra-uterine exposure to antigens and commensal organisms (6), which determine the susceptibility of the newborn to sepsis (6). Notably, our results reveal significant changes in the DNA methylation between septic and controls, indicating that sepsis produces epigenetic reprogramming in leukocytes. With sepsis both hyperinflammatory and immunosuppressive responses occur (6, 41). Our results reinforce this idea, since methylation changes occur simultaneously in both pro- and anti-inflammatory mediators.

Upon infection, the immune response relies primarily on the innate immune system, which is not fully mature in preterm infants. Changes in DNAm were greater in LOS than in EOS when compared to controls. Hence, the lower methylation levels observed in the promoters of genes involved in neutrophil activation (i.e., *ATP8B4, LRG1, TREM1, PRTN3, S100A8, ELANE*, and *CD177*) illustrates the role of DNAm in innate immunity in neonatal sepsis. In addition, TREM-1, which has been proposed as an early biomarker of neonatal sepsis, is involved in the amplification of neutrophil and monocyte inflammatory responses by stimulating pro-inflammatory cytokines (38). Furthermore, the hypomethylation found in the gene coding for neutrophil elastase (*ELANE*) and proteinase 3 (*PRTN3*) may alter the formation of NETs (neutrophil extracellular traps), which is an earlier mechanism used by neutrophils to fight infection (42).

In line with pro-inflammatory responses, previous studies revealed the involvement of CD300b in LPS-induced sepsis (37) as well as apoptotic cell phagocytosis mediated by macrophages (36). As the excessive inflammatory response in sepsis entails apoptosis of lymphocytes and epithelial cells, CD300b contributes to sepsis, so the hypomethylation of the *CD300B* promoter found in our study correlates with previous findings (36, 37). Another relevant pro-inflammatory gene is *LTA* (lymphotoxin alpha), which is a cytokine produced by lymphocytes. This member of the tumor necrosis factor family is associated with increased risk of sepsis in adults (43), so the hypermethylation we found in *LTA* also suggests its use as a predictive marker for risk of sepsis in neonates.

IL-10, together with IL-6 and IL-8, are initial markers with high specificity for neonatal sepsis (32). The upregulation of IL-10 in sepsis is concordant with the lower methylation levels we found in its promoter and may contribute to the immunosuppressive response in neonatal sepsis. In addition, the gene encoding for the inflammatory and immunosuppressive protein S100A8 (S100 calcium binding protein A8) showed low methylation. Interestingly, S100A8 is an alarmin related with immunosuppression, because it prevents the expansion of specific inflammatory monocyte populations in septic neonates (44). Of note, S100A8 participates in neutrophil chemotaxis and adhesion, and it is up-regulated in patients with sepsis (45), which is in agreement with the low methylation levels identified in its promoter found in our study.

Regarding the adaptive immune response, T-cell activation is known to be downregulated during the immunosuppressive state (6). Interestingly, we found that the promoters of genes involved in T-cell regulation (i.e., *CD3D, CD3G, UBASH3A, SIT1*, and *TXK*) were hypermethylated in neonatal sepsis compared to controls, thereby resulting in decreased expression. The CD3D and CD3G proteins are subunits of the TCR-CD3 complex (TCR, T-cell receptor). The TCR-CD3 complex is a multicomponent membrane receptor in mature T cells and in thymocytes, whose under-expression has been described in adult sepsis (46). UBASH3A (part of the T-cell ubiquitin ligand or TULA) and SIT1 participate in the regulation of the TCR-CD3, by negatively regulating TCR signaling (47, 48). The hypermethylation of these genes suggests modulation of the TCR-CD3 complex in neonatal sepsis as well.

Among other significant methylated genes of interest in the context of neonatal sepsis, we also found *CALR*, which codes for calreticulin, an endoplasmic reticulum resident protein, with functions in leukocyte migration and related with the severity of sepsis (49). In addition, our study further revealed the hypomethylation of *MPO* gene, coding for myeloperoxidase, which has previously been described as a potential marker of mortality in sepsis (50).

The overlapping of the DMRs for genes and promoters showed in Figure 4, obtained with mCSEA and DMRcate approaches, revealed the scarce immunoreactivity of T-cells in septic neonates. Interestingly, KEGG pathways demonstrate the enrichment of hypermethylated DMRs for T-cell receptor signaling pathway, Th17 cell differentiation, and PD-L1 expression and PD-1 checkpoint pathway, which has been related with immunosuppression processes (31, 51, 52).

Importantly, using mCSEA method we were able to identify 4 gene promoters in *DUSP22, PM20D1, MIR10A*, and *MIR886* with significant differential DNAm when comparing LOS with EOS. Among these genes, *DUSP22* codes for a JKAP, a JNK pathway-associated phosphatase expressed in various types of human immune cells (e.g., T cells, B cells, and natural killer cells) (33, 53). DUSP22 has been closely related to immune and inflammation response and negatively correlates with APACHE II score, SOFA score, TNF-α, IL-1β, IL-6, and IL-17 (54). It would explain, why IL-6 is found elevated in EOS and considered a clinical criterion to diagnose EOS (55, 56). Interestingly, a work by Khaertynov et al., suggest higher values for TNF-α, IL-1β, IL-6 in LOS than in EOS (57), which is in agreement with the observation of higher methylation of *DUSP22* in LOS than in EOS. In addition, DUSP22 downregulation correlates with severity, higher level of systemic inflammation, and poor survival in adult septic patients (54). It would be interesting to measure DUSP22 methylation in a cohort of neonatal septic patients in order to evaluate its potential as prognostic biomarkers in neonatal sepsis. Regarding *MIR10A*, it has been shown that miR-10a levels in PBMC at admission were significantly lower in sepsis patients compared with non-septic patients with infection, and with healthy controls (58). Moreover, miR-10a levels were found to predict 28-day mortality and negatively correlate with disease severity as well as levels for C-reactive protein, procalcitonin, and inflammatory cytokines such as IL-6, TNF-α, and MCP-1 (58). Regarding *MIR886*, it has been shown that miR-886-5p is increased in human monocyte-derived macrophages as a response to mycobacterial infection at 48 h (59), and the isoform hsa-miR-886-3p was present at higher levels in PBMCs from critically ill patients infected with H1N1 influenza virus than PBMCs from healthy controls (60). Interestingly, miR-886-5p targets IRAK3, BAX, and TP53 transcripts (59), contributing to inhibiting apoptosis (61). In our study we found the hypomethylation of *MIR886* in LOS compared to EOS, which may contribute to its upregulation, thereby inhibiting apoptosis in immune cells and setting the basis for an adaptive response in more mature immune cells. This is of special relevance because persistence of immunosuppression related to epigenetic dysregulation (6) may increase the susceptibility for future infections and increases the risk of mortality (11, 17).

Our study has some limitations, especially in the small number of newborns included in each group. Nonetheless our results importantly set the basis for further research aimed to validate our findings and provide novel biomarkers for neonatal sepsis based on DNA methylation.

## Conclusions

DNAm profiles with differential methylation were observed between newborns with sepsis and control neonates in relevant genes and promoters involved in immune pathways. Our study demonstrates the potential use of DNAm to reveal relevant mechanisms underlying the inflammatory and immune dysregulation in neonatal sepsis, and particularly in EOS and LOS, which can help to clarify its physiopathology and therefore contribute to prevent life-threatening complications during neonatal sepsis.

## Data Availability Statement

The datasets presented in this study can be found in online repositories. The .idat files of the 23 samples were deposited on Gene Expression Omnibus (GEO) with the number GSE155952.

## Ethics Statement

The studies involving human participants were reviewed and approved by Ethics Committee from Hospital Universitario y Politécnico la Fe (Valencia, Spain) with registration number 2017/0470. Written informed consent to participate in this study was provided by the participants' legal guardian/next of kin.

## Author Contributions

JG-G, MV, SM-M, PN, and FP: conception, study design, and coordination. MV, SL-P, and IL-C: wrote the ethics committee application, present the informed consent, and collect the samples. SL-P, PN, MG, IL-C, JB-G, RO-V, and SM-M: experiment performing and data acquisition. PN, MG, and EG-L: statistical analysis. JG-G, SL-P, SM-M, MV, PN, SL-P, EG-L, and FP: drafting manuscript. All authors approved the final version of the manuscript and agreed to be accountable for all aspects of the work in ensuring that questions related to the accuracy or integrity of any part of the work are appropriately investigated and resolved. All persons designated as authors qualify for authorship, and all those who qualify for authorship are listed.

## Conflict of Interest

JG-G, FP, and SM-M are owners of EpiDisease's shares. JG-G and SM-M are currently the C.E.O. and C.S.O. of 1446 EpiDisease S.L., respectively. EpiDisease is a Spin-Off of the Consortium Centre for Biomedical Network Research on Rare Diseases (Spanish Institute of Health- Instituto de Salud Carlos III), the Biomedical Research Centre INCLIVA and the University of Valencia. There are no patents, products in development or marketed products associated with this research to declare. The remaining authors declare that the research was conducted in the absence of any commercial or financial relationships that could be construed as a potential conflict of interest.

## References

[1] Shane AL Sánchez PJ Stoll BJ. Neonatal Sepsis. Lancet. (2017) 390:1770–80. 10.1016/S0140-6736(17)31002-4 28434651

[2] Singer M Deutschman CS Seymour C Shankar-Hari M Annane D. The third international consensus definitions for sepsis and septic shock (sepsis-3). J Am Med Assoc. (2016) 315:801–10. 10.1001/jama.2016.0287 PMC4968574 26903338

[3] McGovern M Giannoni E Kuester H Turner MA van den Hoogen A Bliss JM. Challenges in developing a consensus definition of neonatal sepsis. Pediatr Res. (2020) 88:14–26. 10.1038/s41390-020-0785-x 32126571

[4] Maddux AB Douglas IS. Is the developmentally immature immune response in paediatric sepsis a recapitulation of immune tolerance? Immunology. (2015) 145:1–10. 10.1111/imm.12454 25691226 PMC4405319

[5] Raymond SL Stortz JA Mira JC Larson SD Wynn JL Moldawer LL. Immunological defects in neonatal sepsis and potential therapeutic approaches. Front Pediatr. (2017) 5:14. 10.3389/fped.2017.00014 28224121 PMC5293815

[6] Hibbert JE Currie A Strunk T. Sepsis-induced immunosuppression in neonates. Front Pediatr. (2018) 6:357. 10.3389/fped.2018.00357 30555806 PMC6281766

[7] Cernada M Serna E Bauerl C Collado MC Pérez-Martínez G Vento M. Genome-wide expression profiles in very low birth weight infants with neonatal sepsis. Pediatrics. (2014) 133:e1203–11. 10.1542/peds.2013-2552 24709930

[8] Zea-Vera A Ochoa TJ. Challenges in the diagnosis and management of neonatal sepsis. J Trop Pediatr. (2015) 61:1–13. 10.1093/tropej/fmu079 25604489 PMC4375388

[9] Klinger G Levy I Sirota L Boyko V Reichman B Lerner-Geva L. Epidemiology and risk factors for early onset sepsis among very-low-birthweight infants. Am J Obstet Gynecol. (2009) 201:38.e1-6. 10.1016/j.ajog.2009.03.006 19380122

[10] Benitz WE Achten NB. Technical assessment of the neonatal early-onset sepsis risk calculator. Lancet Infect Dis. (2020) S1473-3099(20) 30490-4. 10.1016/S1473-3099(20)30490-4 33129425

[11] Hedegaard SS Wisborg K Hvas A-M. Diagnostic utility of biomarkers for neonatal sepsis—a systematic review. Infect Dis. (2015) 47:117–24. 10.3109/00365548.2014.971053 25522182

[12] Dong Y Speer CP. Late-onset neonatal sepsis: recent developments. Arch Dis Child Fetal Neonatal Ed. (2015) 100:F257–63. 10.1136/archdischild-2014-306213 25425653 PMC4413803

[13] Strunk T Jamieson SE Burgner D. Genetic and epigenetic susceptibility to early life infection. Curr Opin Infect Dis. (2013) 26:241–7. 10.1097/QCO.0b013e32835fb8d9 23449138

[14] Cross D Drury R Hill J Pollard AJ. Epigenetics in sepsis: understanding Its role in endothelial dysfunction, immunosuppression, and potential therapeutics. Front Immunol. (2019) 10:1363. 10.3389/fimmu.2019.01363 31275313 PMC6591469

[15] Carson WF Cavassani KA Dou Y Kunkel SL. Epigenetic regulation of immune cell functions during post-septic immunosuppression. Epigenetics. (2011) 6:273–83. 10.4161/epi.6.3.14017 21048427 PMC3092675

[16] Goldberg AD Allis CD Bernstein E. Epigenetics: a landscape takes shape. Cell. (2007) 128:635–8. 10.1016/j.cell.2007.02.006 17320500

[17] Berger SL Kouzarides T Shiekhattar R Shilatifard A. An operational definition of epigenetics. Genes Dev. (2009) 23:781–3. 10.1101/gad.1787609 19339683 PMC3959995

[18] Bernstein BE Stamatoyannopoulos JA Costello JF Ren B Milosavljevic A Meissner A. The NIH roadmap epigenomics mapping consortium. Nat Biotechnol. (2010) 28:1045–48. 10.1038/nbt1010-1045 20944595 PMC3607281

[19] Beltrán-García J Osca-Verdegal R Romá-Mateo C Carbonell N Ferreres J Rodríguez M. Epigenetic biomarkers for human sepsis and septic shock: insights from immunosuppression. Epigenomics. (2020) 12:617–46. 10.2217/epi-2019-0329 32396480

[20] Morales-Nebreda L McLafferty FS Singer BD. DNA methylation as a transcriptional regulator of the immune system. Transl Res. (2019) 204:1–18. 10.1016/j.trsl.2018.08.001 30170004 PMC6331288

[21] Saeed S Quintin J Kerstens HHD Rao NA Aghajanirefah A Matarese F. Epigenetic programming of monocyte-to-macrophage differentiation and trained innate immunity. Science. (2014) 345:1251086. 10.1126/science.1251086 25258085 PMC4242194

[22] Lorente-Sorolla C Garcia-Gomez A Català-Moll F Toledano V Ciudad L Avendaño-Ortiz J. Inflammatory cytokines and organ dysfunction associate with the aberrant DNA methylome of monocytes in sepsis. Genome Med. (2019) 11:66. 10.1186/s13073-019-0674-2 31665078 PMC6820973

[23] Binnie A Walsh CJ Hu P Dwivedi DJ Fox-Robichaud A Liaw PC. Epigenetic profiling in severe sepsis: a pilot study of DNA methylation profiles in critical illness. Crit Care Med. (2020) 48:142–50. 10.1097/CCM.0000000000004097 31939781

[24] Dhas DBB Ashmi AH Bhat BV Kalaivani S Parija SC. Comparison of genomic DNA methylation pattern among septic and non-septic newborns—an epigenome wide association study. Genomics Data. (2015) 3:36–40. 10.1016/j.gdata.2014.11.004 26484145 PMC4535661

[25] Töllner U. Early diagnosis of septicemia in the newborn—Clinical studies and sepsis score. Eur J Pediatr. (1982) 138:331–7. 10.1007/BF00442511 7128642

[26] Goldstein B Giroir B Randolph A. International pediatric sepsis consensus conference: definitions for sepsis and organ dysfunction in pediatrics. Pediatr Crit Care Med. (2005) 6:2–8. 10.1097/01.PCC.0000149131.72248.E6 15636651

[27] Aryee MJ Jaffe AE Corrada-Bravo H Ladd-Acosta C Feinberg AP Hansen KD. Minfi: a flexible and comprehensive bioconductor package for the analysis of infinium DNA methylation microarrays. Bioinformatics. (2014) 30:1363–69. 10.1093/bioinformatics/btu049 24478339 PMC4016708

[28] Fortin JP Labbe A Lemire M Zanke BW Hudson TJ Fertig EJ. Functional normalization of 450k methylation array data improves replication in large cancer studies. Genome Biol. (2014) 15:503. 10.1186/s13059-014-0503-2 25599564 PMC4283580

[29] Pidsley R Zotenko E Peters TJ Lawrence MG Risbridger GP Molloy P. Critical evaluation of the illumina MethylationEPIC BeadChip microarray for whole-genome DNA methylation profiling. Genome Biol. (2016) 17:208. 10.1186/s13059-016-1066-1 27717381 PMC5055731

[30] Ritchie M Phipson Wu D Hu Y Law CW Shi W. limma powers differential expression analyses for RNA-sequencing and microarray studies. Nucleic Acids Res. (2015) 43:e47. 10.1093/nar/gkv007 25605792 PMC4402510

[31] Chen X Oppenheim JJ. Th17 cells and Tregs : unlikely allies. J Leukoc Biol. (2014) 95:723–31. 10.1189/jlb.1213633 24563509 PMC3984971

[32] Boskabadi Ha Zakerihamidi M. Evaluate the diagnosis of neonatal sepsis by measuring interleukins: a systematic review. Pediatr Neonatol. (2018) 59:329–38. 10.1016/j.pedneo.2017.10.004 29239828

[33] Zhou R Chang Y Liu J Chen M Wang H Huang M. JNK pathway-associated phosphatase/DUSP22 suppresses CD4+ T-cell activation and Th1/Th17-cell differentiation and negatively correlates with clinical activity in inflammatory bowel disease. Front Immunol. (2017) 8:781. 10.3389/fimmu.2017.00781 28725226 PMC5496234

[34] Peters TJ Buckley MJ Statham AL Pidsley R Samaras K V Lord R. *De novo* identification of differentially methylated regions in the human genome. Epigenetics Chromatin. (2015) 8:6. 10.1186/1756-8935-8-6 25972926 PMC4429355

[35] Breeze CE Reynolds AP Van Dongen J Dunham I Lazar J Neph S. EFORGE v2.0: updated analysis of cell type-specific signal in epigenomic data. Bioinformatics. (2019) 35:4767–69. 10.1093/bioinformatics/btz456 31161210 PMC6853678

[36] Murakami Y Tian L Voss OH Margulies DH Krzewski K Coligan JE. CD300b regulates the phagocytosis of apoptotic cells via phosphatidylserine recognition. Cell Death Differ. (2014) 21:1746–57. 10.1038/cdd.2014.86 25034781 PMC4211373

[37] Yamanishi Y Takahashi M Izawa K Isobe M Ito S Tsuchiya A. A soluble form of LMIR5/CD300b amplifies lipopolysaccharide-induced lethal inflammation in sepsis. J Immunol. (2012) 189:1773–79. 10.4049/jimmunol.1201139 22772446

[38] Benjamini Y Hochberg Y. Controlling the false discovery rate: a practical and powerful approach to multiple testing. J R Stat Soc Ser B. (1995) 57:289–300. 10.1111/j.2517-6161.1995.tb02031.x

[39] Martorell-Marugán J González-Rumayor V Carmona-Sáez P. mCSEA: detecting subtle differentially methylated regions. Bioinformatics. (2019) 35:3257–62. 10.1093/bioinformatics/btz096 30753302

[40] Yu G Wang L-G Han Y He Q-Y. clusterProfiler: an R package for comparing biological themes among gene clusters. Omi A J Integr Biol. (2012) 16:284–7. 10.1089/omi.2011.0118 22455463 PMC3339379

[41] Van Der Poll T Van De Veerdonk FL Scicluna BP Netea MG. The immunopathology of sepsis and potential therapeutic targets. Nat Rev Immunol. (2017) 17:407–20. 10.1038/nri.2017.36 28436424

[42] Delano MJ Ward PA. The immune system's role in sepsis progression, resolution, and long-term outcome. Immunol Rev. (2016) 274:330–53. 10.1111/imr.12499 27782333 PMC5111634

[43] Watanabe E Buchman TG Hirasawa H Zehnbauer BA. Association between lymphotoxin-α (tumor necrosis factor-β) intron polymorphism and predisposition to severe sepsis is modified by gender and age. Crit Care Med. (2010) 38:181–93. 10.1097/CCM.0b013e3181bc805d 19789445 PMC5124381

[44] Heinemann AS Pirr S Fehlhaber B Mellinger L Burgmann J Mandy B. In neonates S100A8/S100A9 alarmins prevent the expansion of a specific inflammatory monocyte population promoting septic shock. FASEB J. (2017) 31:1153–64. 10.1096/fj.201601083R 27993995

[45] Lu X Xue L Sun W Ye J Zhu Z Mei H. Identification of key pathogenic genes of sepsis based on the gene expression omnibus database. Mol Med Rep. (2017) 17:3042–54. 10.3892/mmr.2017.8258 29257295 PMC5783525

[46] Cazalis M-A Lepape A Venet F Frager F Mougin B Vallin H. Early and dynamic changes in gene expression in septic shock patients: a genome-wide approach. Intensive Care Med Exp. (2014) 2:20. 10.1186/s40635-014-0020-3 26215705 PMC4512996

[47] Ge Y Paisie TK Chen S Concannon P. UBASH3A regulates the synthesis and dynamics of TCR–CD3 complexes. J Immunol. (2019) 203:2827–36. 10.4049/jimmunol.1801338 31659016 PMC6938261

[48] Marie-Cardine A Kirchgessner H Bruyns E Shevchenko A Mann M Autschbach F. SHP2-interacting transmembrane adaptor protein (SIT), a novel disulfide- linked dimer regulating human T cell activation. J Exp Med. (1999) 189:1181–94. 10.1084/jem.189.8.1181 10209036 PMC2193021

[49] Xu Z Yang Y Zhou J Huang Y Wang Y Zhang Y. Role of plasma calreticulin in the prediction of severity in septic patients. Dis Markers. (2019) 2019:8792640. 10.1155/2019/8792640 31612071 PMC6757249

[50] Schrijver IT Kemperman H Roest M Kesecioglu J de Lange DW. Myeloperoxidase can differentiate between sepsis and non-infectious SIRS and predicts mortality in intensive care patients with SIRS. Intensive Care Med Exp. (2017) 5:43. 10.1186/s40635-017-0157-y 28916973 PMC5602808

[51] Chen G Huang AC Zhang W Zhang G Wu M Xu W. Exosomal PD-L1 contributes to immunosuppression and is associated with anti-PD-1 response. Nature. (2018) 560:382–6. 10.1038/s41586-018-0392-8 30089911 PMC6095740

[52] Patil N Guo Y Luan L Sherwood E. Targeting immune cell checkpoints during sepsis. Int J Mol Sci. (2017) 18:2413. 10.3390/ijms18112413 29135922 PMC5713381

[53] Chuang H-C Chen Y-M Hung W-T Li J-P Chen D-Y Lan J-L. Downregulation of the phosphatase JKAP/DUSP22 in T cells as a potential new biomarker of systemic lupus erythematosus nephritis. Oncotarget. (2016) 7:57593–605. 10.18632/oncotarget.11419 27557500 PMC5295375

[54] Zhao M Huang X. Downregulation of JKAP is correlated with elevated disease risk, advanced disease severity, higher inflammation, and poor survival in sepsis. J Clin Lab Anal. (2019) 33:e22945. 10.1002/jcla.22945 31206807 PMC6757113

[55] Mirzarahimi M Barak M Eslami A Enteshari-Moghaddam A. The role of interleukin-6 in the early diagnosis of sepsis in premature infants. Pediatr Rep. (2017) 9:7305. 10.4081/pr.2017.7305 29081936 PMC5643948

[56] Krueger M Nauck MS Sang S Hentschel R Wieland H Berner R. Cord blood levels of interleukin-6 and interleukin-8 for the immediate diagnosis of early-onset infection in premature infants. Neonatology. (2001) 80:118–23. 10.1159/000047130 11509811

[57] Khaertynov KS Boichuk S V. Khaiboullina SF Anokhin VA Andreeva AA Lombardi VC Comparative assessment of cytokine pattern in early and late onset of neonatal sepsis. J Immunol Res. (2017) 2017:1–8. 10.1155/2017/8601063 28367457 PMC5357566

[58] Zheng G Qiu G Ge M Meng J Zhang G Wang J. miR-10a in peripheral blood mononuclear cells is a biomarker for sepsis and has anti-inflammatory function. Mediat Inflamm. (2020) 2020:1–10. 10.1155/2020/4370983 32214905 PMC7077053

[59] Sharbati J Lewin A Kutz-Lohroff B Kamal E Einspanier R Sharbati S. Integrated MicroRNA-mRNA-analysis of human monocyte derived macrophages upon *Mycobacterium avium* subsp. hominissuis Infection. PLoS ONE. (2011) 6:e20258. 10.1371/journal.pone.0020258 21629653 PMC3101234

[60] Song H Wang Q Guo Y Liu S Song R Gao X. Microarray analysis of MicroRNA expression in peripheral blood mononuclear cells of critically ill patients with influenza A (H1N1). BMC Infect Dis. (2013) 13:257. 10.1186/1471-2334-13-257 23731466 PMC3679792

[61] Li J-H Xiao X Zhang Y-N Wang Y-M Feng L-M Wu Y-M. MicroRNA miR-886-5p inhibits apoptosis by down-regulating Bax expression in human cervical carcinoma cells. Gynecol Oncol. (2011) 120:145–51. 10.1016/j.ygyno.2010.09.009 20947150

