# Supplementary material for: DNA Methylation Analysis to Unravel Altered Genetic Pathways Underlying Early Onset and Late Onset Neonatal Sepsis. A Pilot Study
Source: Front Immunol. 2021 Feb 15;12:622599. doi: 10.3389/fimmu.2021.622599 (PMC7917190; doi:10.3389/fimmu.2021.622599)
Supplement: Supplementary file 1 [file Data_Sheet_1.docx]

Supplementary Material and Methods

# Experimental design

The study was performed in the Neonatal Intensive Care Unit (NICU) of the Hospital Universitario y Politécnico La Fe (Valencia, Spain) from September 2018 to December 2019. Neonatal sepsis samples from the pilot study entitled “Profile of lipid peroxidation and microRNA as markers of damage to the central nervous system in premature infants with episodes of intermittent hypoxia” were analyzed with a registration number by the CEIM of the institution 2017/0470. Parents of all the patients signed their informed consent.

This is a prospective cohort study that included preterm neonates born at ≤ 32 weeks of gestation age). Inclusion and exclusion criteria for the study are shown in Table 1.

Samples were collected at 5-7 days after birth. A total of 23 patients were analyzed. Patients who met late onset (LOS) and early onset (EOS) sepsis criteria were included in the study (1,2). Nine patients had confirmed LOS defined as sepsis occurring >72 hours after birth, six patients had confirmed EOS defined as occurring <72 hours after birth, and two patients suffered both types. Six non-septic preterm neonates were used as controls.

The diagnosis of EOS required a positive peripheral smear culture, suggestive symptoms (Table 1), and/or increase of plasma C-reactive protein or IL-6. LOS diagnosis required a positive blood culture or suggestive clinical symptoms. These criteria were not mutually exclusive and some patients were identified as suffering from both types of sepsis.

We want to indicate that LOS patients are more willingness to acquire nosocomial sepsis, which is much more common in newborn infants with lower gestational ages because they have an immature immunological response, stay long in the hospital, are submitted a significantly greater number of invasive intervention (indwelling catheters, endotracheal intubation, mechanical ventilation, parenteral nutrition, analytical procedures, etc.) Therefore, babies with LOS have less weight and less gestational age.

Babies can have an early onset sepsis acquired during late gestation or during birth, and thereafter along hospitalization they can acquire a late onset sepsis caused by a nosocomial bacteria.

# DNA methylation profiling using Illumina EPIC 850K array

The measurement of genome-wide methylation on the 23 samples was performed by means of the Infinium Human DNA Methylation EPIC 850K arrays (Illumina Inc, San Diego, CAL, USA) which interrogates over 850,000 CpG sites across the genome.

First, the genomic DNA was treated with bisulfite, followed by a whole genome amplification step, enzymatic endpoint fragmentation, precipitation, and resuspension. The processed samples were then hybridized (at 48 °C for 16 hours) on the bead array. Afterwards, unhybridized and non-specifically bound DNA was washed away, and a single nucleotide extension was performed using nucleotides labelled with biotin (ddCTP and ddGTP) and 2,4-dinitrophenol (ddATP and ddTTP). Repeated rounds of staining were performed with a combination of antibodies that differentiate DNP and biotin by fixing them with different fluorophores. Finally, the BeadChip was washed and protected to scan it on an Illumina HiScan SQ scanner (Illumina Inc., San Diego, CA, USA).

# Bioinformatic analysis detailed information

The minfi R-package (3) was used for the reading of the intensity files, as well as quality assessment, functional normalization between arrays (4), and filtering of probes attending to poor detection p-value (<0.01) in any of the samples, probes with common SNPs, probes located at the sexual chromosomes (X & Y), and cross-reactive probes (5).

The identification of DMCs (differentially methylated CpGs) was performed considering an FDR cutoff of 0.05 using limma. With the aim of discovering DMRs, we employed two complementary bioinformatic tools: DMRcate (6) and mCSEA (7) R packages. We performed the differential analysis between the following clinical groups: neonatal sepsis patients versus the control group, neonatal sepsis of nosocomial origin versus control individuals, vertical sepsis versus control individuals, and nosocomial sepsis versus vertical sepsis. The parameters used for the implementation of DMRcate were a lambda of 1,000 nucleotides and a scaling factor for bandwidth of 2. Regarding mCSEA, the significance threshold to retrieve the DMRs was defined as an FDR<0.05. Finally, we performed the overlap of both DMR sets.

The sets of DMRs were functionally enriched in both GO terms and KEGG pathways by means of an over-representation analysis (ORA) using the clusterProfiler R-package (8) allowing identification of the biological processes and metabolic pathways that may be altered by differential methylation levels.

Furthermore, the top 1,000 DMCs were analyzed for cell-type enrichment using eFORGE (9), which tests for overlapping with epigenetic tracks revealing cell types and regulatory element classes (promoters, enhancers, and transcribed regions) in which the CpGs are enriched.

All computational steps, except for the eFORGE analysis, were performed using in-house R scripts.

# References

1. Töllner U. Early diagnosis of septicemia in the newborn - Clinical studies and sepsis score. *Eur J Pediatr* (1982) **138**:331–337. doi:10.1007/BF00442511

2. Goldstein B, Giroir B, Randolph A. International pediatric sepsis consensus conference: Definitions for sepsis and organ dysfunction in pediatrics. in *Pediatric Critical Care Medicine* (Pediatr Crit Care Med), 2–8. doi:10.1097/01.PCC.0000149131.72248.E6

3. Aryee MJ, Jaffe AE, Corrada-Bravo H, Ladd-Acosta C, Feinberg AP, Hansen KD, Irizarry RA. Minfi: A flexible and comprehensive Bioconductor package for the analysis of Infinium DNA methylation microarrays. *Bioinformatics* (2014) **30**:1363–1369. doi:10.1093/bioinformatics/btu049

4. Fortin JP, Labbe A, Lemire M, Zanke BW, Hudson TJ, Fertig EJ, Greenwood CMT, Hansen KD. Functional normalization of 450k methylation array data improves replication in large cancer studies. *Genome Biol* (2014) **15**:503. doi:10.1186/s13059-014-0503-2

5. Pidsley R, Zotenko E, Peters TJ, Lawrence MG, Risbridger GP, Molloy P, Van Djik S, Muhlhausler B, Stirzaker C, Clark SJ. Critical evaluation of the Illumina MethylationEPIC BeadChip microarray for whole-genome DNA methylation profiling. *Genome Biol* (2016) **17**:208. doi:10.1186/s13059-016-1066-1

6. Peters TJ, Buckley MJ, Statham AL, Pidsley R, Samaras K, V Lord R, Clark SJ, Molloy PL. De novo identification of differentially methylated regions in the human genome. *Epigenetics and Chromatin* (2015) **8**: doi:10.1186/1756-8935-8-6

7. Martorell-Marugán J, González-Rumayor V, Carmona-Sáez P. mCSEA: detecting subtle differentially methylated regions. *Bioinformatics* (2019) **35**:3257–3262. doi:10.1093/bioinformatics/btz096

8. Yu G, Wang L-G, Han Y, He Q-Y. clusterProfiler: an R Package for Comparing Biological Themes Among Gene Clusters. *Omi A J Integr Biol* (2012) **16**:284–287. doi:10.1089/omi.2011.0118

9. Breeze CE, Reynolds AP, Van Dongen J, Dunham I, Lazar J, Neph S, Vierstra J, Bourque G, Teschendorff AE, Stamatoyannopoulos JA, et al. EFORGE v2.0: Updated analysis of cell type-specific signal in epigenomic data. *Bioinformatics* (2019) **35**:4767–4769. doi:10.1093/bioinformatics/btz456

# Supplementary Tables

Supplementary Table 1. Inclusion and exclusion criteria

| **Inclusion criteria** | **Exclusion criteria** |
| --- | --- |
| - Temperature instability (> 38 ºC or < 36.5 ºC) - Respiratory symptoms (respiratory distress, apnea, or cyanosis) - Cardiovascular symptoms (hypotension, tachycardia, bradycardia, or poor perfusion) - Neurological symptoms (clinical or electrical seizures, hypotonia, or lethargy) - Gastrointestinal symptoms (vomiting, poor feeding, feeding intolerance, or abdominal distension) - Suspicion of sepsis | - Intraventricular hemorrhage grades III-IV - Chromosomal abnormalities - Major malformation - Seizures - Surgery 72 hours before recruitment - Persistent ductus arteriosus requiring treatment at the time of recruitment - Invasive mechanical ventilation) |

# Supplementary Figures

**Supplementary Figure 1**. Representation of the percentatge of microorganisms the identified in the blood culture for EOS, LOS and LOS+EOS cases.
